# How does family functioning contribute to academic-related outcomes of Chinese adolescents: the mediating role of spirituality

**DOI:** 10.3389/fpsyg.2024.1357473

**Published:** 2024-06-03

**Authors:** Daniel T. L. Shek, Kim Hung Leung, Xiang Li, Diya Dou, Xiaoqin Zhu

**Affiliations:** Department of Applied Social Sciences, The Hong Kong Polytechnic University, Kowloon, Hong Kong SAR, China

**Keywords:** family functioning, spirituality, academic values, academic anxiety, mediator, Chinese adolescents

## Abstract

While family functioning is crucial to adolescent developmental outcomes, the mediating role of spirituality in the relationship between family functioning and academic-related outcomes of adolescents has been sparsely explored, particularly in non-Western contexts. To address this gap, based on a short-term longitudinal study, we examined the influence of family functioning on the academic values and academic anxiety of 4,981 Chinese adolescents in Sichuan, China, with spirituality as the mediator. We gathered data from students aged 11 and above at Wave 1 and at six months later (Wave 2). Analysis utilizing structural equation modeling indicated that prior family functioning positively and negatively predicted subsequent academic values and academic anxiety respectively, with spirituality as a significant mediator. Theoretically, this study helps to build up a conceptual model on how family functioning and spirituality of adolescents shape academic values and academic anxiety of adolescents. Practically, the present findings highlight the significance of enhancing family functioning and adolescent spirituality to help adolescents strive for academic success.

## Introduction

Formal education is a way to cultivate knowledge and competency of people in addition to maintenance of harmony and betterment of the society (Govindarajoo et al., 2022). As such, enhancement of students’ school performance is of paramount importance. In particular, academic values are significant determinants of academic outcomes (Jiang et al., 2020; Li et al., 2021). Past studies have shown that examination scores, school belongingness, engagement in learning, satisfaction with subjects and academic performance, academic self-concept, and psychological well-being of students are related to their academic values (e.g., Katrin et al., 2019; Jiang et al., 2020; Dou and Shek, 2021; Li et al., 2021; Wang et al., 2021; Allen et al., 2023). Nevertheless, striving for academic excellence would create much learning stress and academic anxiety to students (Xu et al., 2022). Previous research has showed that academic anxiety is negatively related to learning ability, self-confidence, academic performance, and positively predicted smartphone addiction, academic burnout, negative mood, and self-handicapping behaviors (e.g., Jia et al., 2021; Adams et al., 2022; Wu et al., 2022; Xu et al., 2022; Zeng et al., 2022). As a consequence, academic anxiety undermines students’ academic performance.

Different models have highlighted the role of both individual and contextual factors in academic values and academic anxiety of adolescents. According to Eccles et al.’s (1983) expectancy-value model of achievement motivation, students’ academic performance and choices are determined by their success expectancy as well as values of academic tasks. The subjective value that adolescents place on their academic tasks are affected by both individual (e.g., perceived competencies) and environmental factors (e.g., school environment). Similarly, in Cassady’s (2022) emotional processing model of academic anxiety, academic anxiety is triggered by different personal (e.g., perfectionism) and contextual (e.g., high stakes exams) antecedents. There are other models focusing on ecological systems (Bronfenbrenner, 1974; Sales and Irwin, 2013). Amongst different social contexts of adolescent development, the influence of the family in adolescent development has been highlighted (e.g., Hayek et al., 2022; Tamayo-Aguledo et al., 2022; Shek et al., 2022a).

With reference to China, research findings have illustrated that both personal factors, such as achievement goal orientation (Lin et al., 2021) and feeling of enjoyment (Yuan, 2023), as well as social contextual factors such as family socioeconomic status (Yeung et al., 2022) and parental control (Xu et al., 2021) contribute to academic values and academic anxiety of Chinese adolescents. However, most studies adopt a cross-sectional approach. The relationships amongst family factors, adolescents’ personal attributes and academic values and academic anxiety of adolescents are not systematically examined. In response to these research gaps, we examined how family functioning is related to adolescent academic values and academic anxiety, with spirituality proposed as a mediator.

### Family functioning and adolescent academic values

Obviously, family functioning is an important context for adolescent development (Folk et al., 2020; Ma et al., 2021). Different family functioning models such as family systems theory (Cox and Paley, 1997) and Coleman’s social capital model (Coleman, 1988) support the idea that positive family functioning is vital to educational attainment of adolescents (Ma et al., 2021; Puente, 2022). With particular reference to China, family functioning factors, especially parental effort, are regarded as crucial determinants of academic success of Chinese adolescents (Jin, 2023).

With reference to Eccles et al.’s (1983) achievement motivation model, parental beliefs, expectations, attitudes and behaviors are important family processes shaping the academic values of adolescents. For example, Puente (2022) investigated the relationships between family support and academic values in 3,060 Latinx adolescents, and she found that parental support (such as conversation, emotional support and coactivity) contributed to the academic values of adolescents. Rozek et al. (2015) assessed the impact of STEM utility-value intervention for 188 USA parents on future STEM values and STEM course-taking of their children over time. Results showed that the increase in mothers’ STEM utility value promoted those of adolescents. In contrast, except a few exceptions (e.g., Bi et al., 2020), research on the impact of family functioning on Chinese adolescents’ academic values is almost non-existent. After searching with PsycINFO database utilizing “family functioning” and “academic values” in ‘Any Field’ in April 2024, we did not find any record of publication. As such, there is no investigation to examine the direct impact of systemic family functioning on academic values of Chinese adolescents.

### Family functioning and adolescent academic anxiety

Apart from the educational environment (Tripon et al., 2023), family is also an important foundation of adolescent psychological well-being (e.g., Li et al., 2022; van Eickels et al., 2022). Different family functioning models such as Cox and Paley’s (1997) family systems theory and Conger et al.’s (1994) family stress model of economic hardship support the conjecture that favorable family functioning promotes the well-being of adolescents (Stewart et al., 2022; Zhu et al., 2022).

Regarding the family environment model of test anxiety, family communication and structures, relationship amongst family members, and encouragement for personal growth are key determinants of academic anxiety of adolescents (Peleg-Popko and Klingman, 2002). Past research has also revealed an inverse correlation between family functioning and academic anxiety of adolescents (e.g., Trikoilis, 2023; Zanabazar et al., 2023).

Again, except a few studies (e.g., Ma et al., 2021; Xu et al., 2021; Chen et al., 2023), there are few studies on the impact of family functioning on academic anxiety of Chinese adolescents.

### Spirituality and adolescent academic values

Spirituality is viewed as individual’s personal search for getting answers to the questions on the meaning of life and the relationship with the scared or transcendent (King and Boyatzis, 2015; Shek et al., 2021). Past research has showed that spirituality and academic performance of students are positively correlated (e.g., Cooper et al., 2020; David et al., 2022). However, despite there is association between academic performance and academic values of students (e.g., Liu et al., 2020; Meng and Hu, 2023), the relationship between spirituality and academic values of adolescents is rarely explored, except in a few studies (e.g., Ariani, 2021). In view of close conceptual overlap between academic values and academic motivation of students (see Urhahne and Wijnia, 2023), we hypothesized a positive association between spirituality and academic values of adolescents in the present study. To our best knowledge, no study has to date been performed to investigate the direct impact of spirituality on academic values of adolescents in mainland China.

### Spirituality and adolescent academic anxiety

In addition to academic motivation, spirituality also promotes well-being of adolescents (e.g., Nimmi et al., 2021; Shroff et al., 2023). According to Lazarus and Folkman’s (1984) theory of stress and coping, spirituality is seen as a vital way to help adolescents cope with their academic anxiety (Ekwonye et al., 2020). It is because spiritual practices would mitigate negative emotions adolescents experienced in stressful circumstances or help adolescents think about the stressful situations with greater sense of clarity. However, the influence of religiosity, instead of spirituality, on academic anxiety of adolescents has only been examined to date (Yusdiana et al., 2019). After checking with the PsycINFO database utilizing “spirituality” and “academic anxiety” in ‘Any Field’ in April 2024, we did not find any record of publication.

### Spirituality as a mediator

Consistent with the ecological model, previous research has illustrated that spirituality (personal factor) and family functioning (contextual factor) are correlated with each other. For instance, Holmes (2023) found discussing faith in Christian families significantly predicted faith of children.

Lubis and Hasanuddin (2023) also reported positive contribution of family functioning to adolescent psychosocial competence including spirituality. As such, we expected that family functioning contributed positively to the development of spirituality of adolescents in this study.

To our best knowledge, there is no research on the impact of family functioning on academic values and academic anxiety of adolescents with spirituality as a mediator. There are only very few related studies. Stokes (2008) found that parental religiosity and co-attendance positively contributed to academic success of students via students’ spiritual experience. In view of the close association of religiosity and spirituality (see Hill and Pargament, 2003), we hypothesized that family functioning would contribute to academic values of adolescents via adolescents’ spirituality. In addition, Wahyuni et al. (2020) investigated the influence of the family on mental health of college students. The findings revealed that family support was a significant predictor of students’ mental health. Also, they indicated the mediating role of students’ spiritual experience in the influence of the family on mental health of students. Based on these findings, we hypothesized that family functioning would affect academic anxiety of adolescents via adolescents’ spirituality.

There are several limitations of the studies in this area First, research on the contributions of family functioning and spirituality to academic values and academic anxiety of adolescents is very limited. Second, there is no research on the mediating role of spirituality in family functioning - academic values and family functioning - academic anxiety. Third, very few studies in this field have been carried out longitudinally. Lastly, the number of participants in some studies is small (e.g., Qin et al., 2021; Xu et al., 2021), thus rendering the generalization problem of research findings.

### Research objectives and questions

To address these inadequacies, this study aimed at exploring the relationships between family functioning and spirituality, and academic values and academic anxiety of Chinese adolescents. In addition, the mediating effect of spirituality on the paths from family functioning to academic values and academic anxiety were examined. Research questions in the present study were stated as follows:

*Question 1*: Does family functioning predict adolescent academic values? With reference to Eccles et al.’s (1983) model of achievement motivation and previous studies (Rozek et al., 2015; Puente, 2022), our first hypothesis is that family functioning would positively predict adolescent academic values.

*Question 2*: Does family functioning predict adolescent academic anxiety? According to Peleg-Popko and Klingman’s (2002) family model of test anxiety and past studies (Chen et al., 2023; Trikoilis, 2023), our second hypothesis is that family functioning would inversely predict adolescent academic anxiety.

*Question 3*: Does family functioning contribute to adolescent spirituality? Based on the notion by King and Boyatzis (2015), our third hypothesis is that family functioning contributed to adolescent spirituality in a positive way.

*Question 4*: Does spirituality contribute to adolescent academic values? According to Demerouti et al.’s (2001) Job Demands-Resources model and Ariani’s (2021) study, our fourth hypothesis is that spirituality positively contributed to adolescent academic values.

*Question 5*: Does spirituality contribute to adolescent academic anxiety? Based on Lazarus and Folkman’s (1984) theory of stress and coping, and Yusdiana et al.’s (2019) study, our fifth hypothesis is that spirituality contributed to adolescent academic anxiety in a negative way.

*Question 6*: Does spirituality mediate the link between family functioning and academic values of adolescents? Based on Bronfenbrenner’s (1974) ecological systems theory and Stokes’s (2008) study, our sixth hypothesis is that there was significant mediating effect of spirituality in the link from family functioning to adolescent academic values.

*Question 7*: Does spirituality mediate the link from family functioning to adolescent academic anxiety? Based on Bronfenbrenner’s (1974) ecological systems theory and Wahyuni et al.’s (2020) study, our seventh hypothesis is that there was significant mediating effect of spirituality in the link from family functioning to adolescent academic anxiety.

## Materials and methods

### Participants

At the beginning of the survey (Wave 1), there were 5,690 students participated in this study. They were recruited from five high schools in Sichuan, China utilizing the cluster sampling method in 2019. After six months (Wave 2), 4,981 students answered the same questionnaires again (attrition rate = 12.5%). As a result, the responses from two waves of 4,922 students were successfully matched. In the matched sample, mean age of students was 13.1 years old (SD = 1.32). 51.5% of students were female while 48.5% were male. 99.3% of students were Hans. As research questions focused on adolescents, we only analyzed the responses of students aged 11 and above.

### Instruments

This survey involved several valid and reliable scales to examine psychosocial health of adolescents. Consistent with the objective of this study, family functioning, spirituality, academic values in addition to academic anxiety were foci variables in the present study.

### Family functioning

We used a 33-item Chinese Family Assessment Instrument (C-FAI) to examine adolescents’ perceptions on their family functions (Shek, 2002) in terms of mutuality, communication, conflict and harmony, parental concern, as well as parental control. Students were required to rate each item by choosing one of five response options along most similar (1) to most dissimilar (5). To equate higher item scores with better family functioning, all responses of positive items were reverse-coded. C-FAI has been found to be valid and reliable in previous research utilizing Chinese adolescents (e.g., Shek et al., 2022a,b, 2023). In this study, both first-order and second-order factor structures of family functioning were empirically supported. The CFA results of this study were the same as those in Shek et al.’s (2022a,b) study as the dataset used was the same for this study and Shek et al.’s ones. In sum, five primary factors and a higher-order factor of the C-FAI have been identified. All subscales possess acceptable internal reliability. Kindly note that the RMSEA and SRMR values reflect fair fit of the C-FAI to the data, despite CFI and NNFI indices revealed excellent fit.

### Spirituality

We utilized the spirituality scale of The Chinese Positive Youth Development Scale (CPYDS, Shek and Ma, 2010) to examine adolescent spirituality. CPYDS is a validated instrument to assess 15 positive attributes of adolescents such as bonding and self-efficacy. Among all positive youth attribute scales, a 7-item spirituality scale was utilized in this study to examine students’ perceptions on their lives (e.g., My life is empty versus full of excitement) on a 7-point scale (1 = most negative; 7 = most positive). The item score was positively correlated with level of spirituality of adolescents. CPYDS, with particular reference to spirituality scale, has been revealed to possess sound psychometric properties (e.g., Zhu and Shek, 2020; Yu and Shek, 2021). The present CFA results provided empirical support to the unidimensionality of the spirituality scale (*χ*^2^ (df) = 478.52 (14), NNFI and CFI > 0.97, RMSEA <0.085). Factor loadings were greater than 0.59 and all significant at 0.05 level. The reliability of spirituality scale was good as both composite reliability and Cronbach’s alpha values were greater than 0.90 and the mean inter-item correlation was more than 0.60.

### Academic values

Perceived academic values of adolescents were assessed by Students’ Academic Values Scale (Guo et al., 2017) which examined how adolescents view the schoolwork as interesting and to what extent that adolescents like their schoolwork (intrinsic value), as well as perceived usefulness of the schoolwork right now and to the future (utility value). Students were required to answer two items for their perceived intrinsic value (e.g., To what extent do you like doing your schoolwork) while three items for utility value of their study (e.g., To what extent do you find things you have learnt in school to be useful in your daily life). Past research with Chinese adolescents as samples has shown that the scale for assessing academic values is valid and reliable (e.g., Dou and Shek, 2021). The CFA results of this study supported the two-factor correlated model of the Academic Values Scale (*χ*^2^ (df) = 163.25 (4), NNFI and CFI > 0.97, RMSEA <0.090). The range of the loadings was 0.64 to 0.91 (*p* < 0.05). The reliability of all subscales were good (composite reliability >0.85 and all AVEs >0.50) and high correlation coefficient (*r*) among two factors (= 0.72). Likewise, the present findings confirmed the second-order factorial model of the Scale (*χ*^2^ (df) = 163.3 (3), NNFI and CFI > 0.95). Cronbach’s alpha of the Scale was 0.89. Kindly note that the RMSEA value (= 0.10) illustrates fair fit of the Scale to the data, although CFI and NNFI indices revealed excellent fit

### Academic anxiety

In this study, academic anxiety of adolescents was assessed by three items with different response options, including “How worried are you about lagging behind in your studies?” (1 = not worry at all; 5 = very worry), “How nervous are you when the teacher hands out the graded papers?”, and “How nervous are you going to be during the examination?” (1 = not nervous at all; 5 = very nervous). Li et al. (2022) has found this scale was a valid measure to academic anxiety of Chinese adolescents. The present CFA results supported the unidimensional model of academic anxiety scale (*χ*^2^ = 0.14, df = 1, *p* = 0.71; NNFI = CFI = 1.00, RMSEA = 0.000, SRMR = 0.001). Factor loadings were significant (*p* < 0.05) with the range between 0.67 and 0.83. The reliability of academic anxiety scale was good as composite reliability and Cronbach’s alpha values of the scale were 0.78 and 0.77, respectively, and the average inter-item correlation was 0.53.

### Procedure

Before the survey, an ethics approval for investigation was obtained from the Ethics Committee of Sichuan University (Approval code: K2020025; Approval date: 31 July 2020). Besides, informed consent to participate in the survey was got from parents and their students, and schools (see Dou et al., 2021 for details of the survey). Afterwards, the scales were administered to all participants (Wave 1). Key principles for data collection and usage such as anonymity and confidentiality were clearly explained to the students before gathering the data. After six months (Wave 2), students were asked to answer the same questionnaires again. Responses from Wave 1 and Wave 2 were matched and analyzed subsequently.

### Data analysis

**Figure 1:**
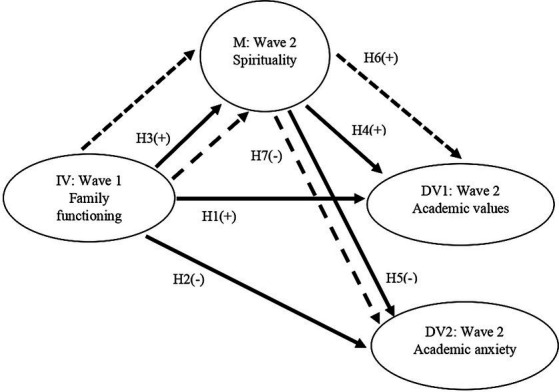
Conceptual model for the mediating effect of Wave 2 spirituality on the paths from Wave I family functioning to academic values and academic anxiety at Wave 2. The solid line represents the direct effect from the antecedent variable to the outcome variable (HI to H5). The dash line represents the mediating path from Wave I family functioning to academic values (H6) and academic anxiety (H7) at Wave 2, with Wave 2 spirituality as the mediator. IV = independent variable, M = mediator, DV = dependent variable, (+) = expected positive relationship, (−) = expected negative relationship.

Figure 1 illustrates the conceptual model of this study. It was utilized to assess whether spirituality mediates the impact of family functioning on academic values and academic anxiety of Chinese adolescents. Before testing this model, we assessed three basic conditions for the mediational analysis (see Baron and Kenny, 1986). These include significant correlations between (1) family functioning and spirituality, (2) spirituality and academic values as well as anxiety, when regressing academic values and academic anxiety on both family functioning and spirituality, and (3) family functioning and academic values as well as academic anxiety of adolescents.

After confirming three basic conditions for the development of mediational models of academic values and academic anxiety, we tested the measurement part of the overall SEM model (see Weston and Gore, 2006). In the measurement model, latent constructs of family functioning, spirituality, academic values, and academic anxiety were specified as freely correlated with each other without any correlated errors among the indicators. If the measurement model fits the data well, we proceed further to examine the structural part of the overall SEM model that has specified different predictions among the constructs.

All estimates in the overall SEM model were obtained by using LISREL 8.54 with maximum likelihood estimation. As stated by Brown (2006), the model fits the data when standardized root-mean-square residual and root-mean-square error of approximation were less than 0.060, and non-normed fit index and comparative fit index were more than 0.95. As large sample was utilized in this study, the error of parameter estimation becomes smaller. Sobel test could be adopted to assess the significance of indirect effects (Özdil and Kutlu, 2019), in which the presence of zero within 95% confidence intervals for the estimates implies non-significance (Lau and Cheung, 2012).

Moreover, confounding effect of age and gender were controlled for in the overall SEM model by specifying the relationship between them and spiritualty, academic values, and academic anxiety at Wave 2 (see Little, 2013). This step is important to reduce the bias of the study since past research has revealed significant contribution of age and gender to spirituality, academic values, and academic anxiety of adolescents. (e.g., Lee et al., 2019; Xie et al., 2019; Schweder and Raufelder, 2024). In fact, multiple regression analyses of this study revealed significant contribution of age and gender to spirituality (age: *β* = −0.13; gender: *β* = 0.10), academic values (age: *β* = −0.23), and academic anxiety (gender: *β* = −0.08) (*p* < 0.001).

## Results

### Descriptive statistics

#### Attrition analyses

The findings from sample attrition analysis over six months illustrated that selective attrition was unlikely to happen in this study. It was because only 12.5% of Wave 1 participants withdrew from the study at Wave 2, with the number of dropouts was not very large. Besides, demographic features of students, such as age and sex, in the matched sample and dropouts was similar to each other. In addition, attrition bias was less likely to happen since the result of the Little’s MCAR test (*χ*^2^_(141)_ = 63.10, *p* = 1.00) indicated complete randomness in missing values of the dataset (see Asendorpf et al., 2014).

#### Descriptive statistics, normality, reliability, and inter-correlations of study variables

Table 1 reports that the mean of all study variables except age ranged from 3.30 to 5.50 with standard deviation in-between 0.73 to 1.40. Based on Finney and DiStefano (2006), the distribution of all item scores was normal since the absolute skewness and kurtosis values of all items were not greater than 2 and 7, respectively. All scales were reliable as the value of Cronbach’s alpha were more than 0.70 (see Cheung et al., 2023) As expected, there were positive associations between family functioning and spirituality, and academic values and its dimensions (intrinsic value and utility value). Moreover, academic values and its dimensions were inversely associated with academic anxiety of adolescents. In addition, family functioning and spirituality were associated with each other in a positive way.

**Table 1:** Descriptive statistics, reliability, and correlations among the variables (*N* = 4,922).

	1	2	3	4	5	6	7	8	9	10	11	12	13
1. Age	–												
2. Sex^a^	0.00	–											
3. W1 MU	**−0.13**	0.00	–										
4. W1 COM	**−0.15**	−0.03	**0.83**	**–**									
5. W1 CONF	**−0.10**	**0.04**	**0.50**	**0.44**	**–**								
6. W1 PCONC	**−0.06**	**0.08**	**0.65**	**0.58**	**0.52**	**–**							
7. W1 PCONT	**−0.05**	**0.08**	**0.29**	**0.31**	**0.54**	**0.39**	**–**						
8. W1 FF	**−0.12**	**0.05**	**0.83**	**0.80**	**0.77**	**0.80**	**0.69**	**–**					
9. W2 SP	**−0.13**	**−0.10**	**0.32**	**0.36**	**0.27**	**0.23**	**0.25**	**0.37**	**–**				
10. W2 INT	**−0.19**	−0.03	**0.25**	**0.28**	**0.19**	**0.13**	**0.16**	**0.26**	**0.52**	**–**			
11. W2 UTIL	**−0.23**	0.01	**0.24**	**0.26**	**0.25**	**0.20**	**0.19**	**0.29**	**0.51**	**0.64**	**–**		
12. W2 AVALUE	**−0.23**	−0.01	**0.27**	**0.29**	**0.25**	**0.18**	**0.20**	**0.31**	**0.57**	**0.89**	**0.92**	–	
13. W2 AANX	−0.01	**0.08**	**−0.07**	**−0.08**	**−0.12**	**−0.07**	**−0.11**	**−0.12**	**−0.16**	**−0.07**	−0.02	**−0.05**	–
Mean	13.14	–	4.18	4.04	3.97	4.37	3.84	4.08	5.50	3.51	4.16	3.90	3.30
SD	1.32	–	0.87	0.96	0.84	0.91	1.14	0.73	1.40	0.96	0.75	0.76	1.09
α	–	–	0.93	0.91	0.69	0.69	0.80	0.95	0.93	0.88	0.89	0.89	0.77
Mean inter-item correlation	–	–	0.53	0.53	0.28	0.45	0.57	0.36	0.65	0.78	0.73	0.62	0.53

Standardized estimates in bold form are significant at 0.01 levels. MU = mutuality, COM = communication, CONF = conflict and harmony, PCONC = parental concern, PCONT = parental control, FF = family functioning, SP = spirituality, AVALUE = academic values, INV = intrinsic value, UTIL = utility value, AANX = academic anxiety, W1 = Wave 1, W2 = Wave 2.

#### Predictive effects of family functioning and spirituality on adolescent academic values and academic anxiety

Table 2 shows the significant family functioning–spirituality, family functioning–academic values, and spirituality–academic values linkages (*p* < 0.05) when each linkage was assessed individually. Similar linkages for using academic anxiety instead of academic values were also found. As such, it was reasonable to test the mediating role of spirituality in-between family functioning and academic values and academic anxiety of adolescents (see Baron and Kenny, 1986).

**Table 2:** Conditions for the establishment of mediational models.

Pathways	Standardized effects	*χ* ^2^	df	*p-*value	NNFI	CFI	RMSEA	SRMR
*1. For academic values*								
W1 Family functioning ➔ W2 Spirituality	0.37	24714.3	731	<0.001	0.95	0.95	0.100	0.090
W1 Family functioning ➔ W2 Academic values	0.33	24374.6	654	<0.001	0.94	0.95	0.110	0.095
W2 Spirituality ➔ W2 Academic values (after controlling for W1 Family functioning)	0.62	26548.3	933	<0.001	0.95	0.95	0.095	0.094
*2. For academic anxiety*								
W1 Family functioning ➔ W2 Spirituality	0.37	24714.3	731	<0.001	0.95	0.95	0.100	0.090
W1 Family functioning ➔ W2 Academic anxiety	−0.08	23833.9	586	<0.001	0.94	0.94	0.120	0.100
W2 Spirituality ➔ W2 Academic anxiety (after controlling for W1 Family functioning)	−0.17	24991.4	850	<0.001	0.95	0.95	0.100	0.090

All effects were significant at the 0.05 level.

#### Mediating effects of spirituality

**Figure 2:**
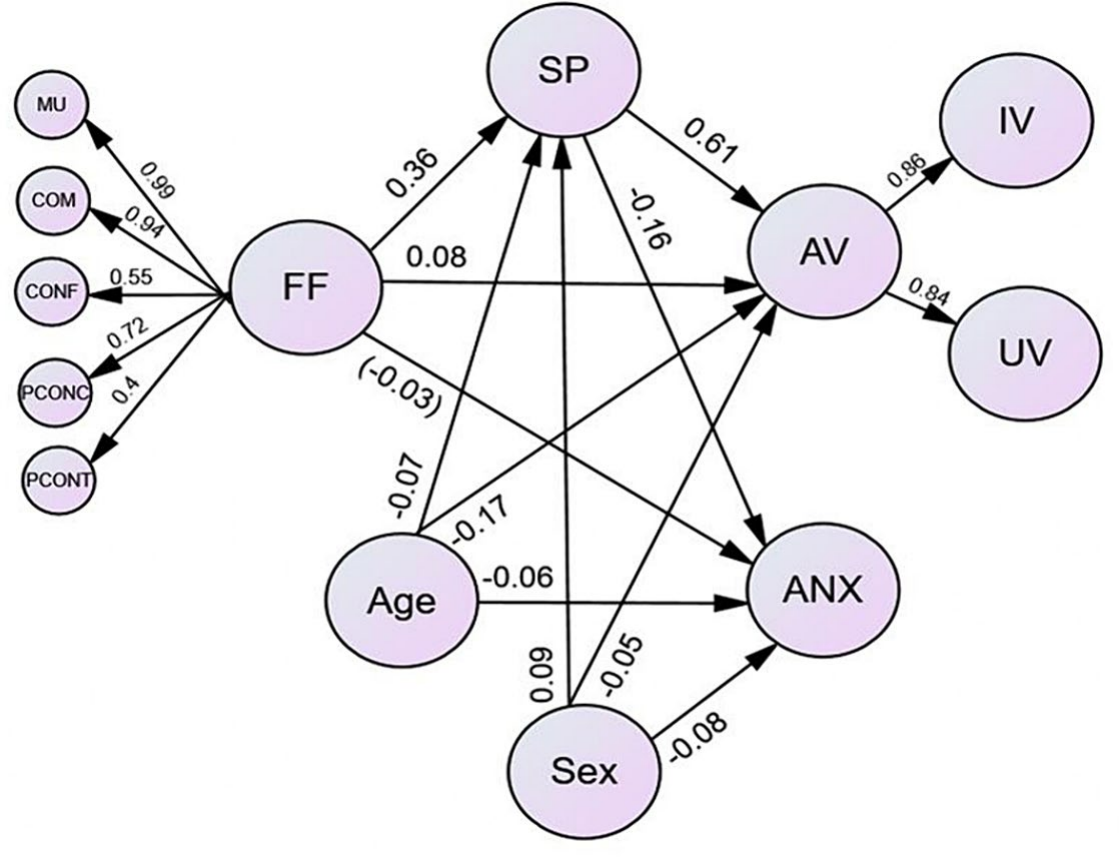
Mediation model of Wave 2 spirituality on the path from Wave I family functioning to Wave 2 academic value and academic anxiety while controlling for age and sex. Standardized estimates are shown in the figure. Except for the path with the estimate in parenthesis, all paths were significant (*p* < 0.05). For clarity, indicators of all constructs and inter-factor correlations of family functioning and academic values are omitted. MU = mutuality, COM = communication, CONF = conflict and harmony, PCONC = parental concern, PCONT = parental; control, FF—family functioning, SP = spirituality, AV = academic values, IV—intrinsic value, value, ANX = academic anxiety.

The measurement portion of the overall SEM model fitted the data in a satisfactory manner in this study (*χ*^2^ (df) = 30335.5 (1068), *p* < 0.001, NNFI and CFI = 0.95, RMSEA = 0.09, SRMR = 0.11). Likewise, the structural portion fitted the data well (*χ*^2^ (df) = 26288.5 (1155), *p* < 0.001, CFI = 0.96, NNFI = 0.95, RMSEA = 0.085, SRMR = 0.077). In particular, family functioning at Wave 1 contributed to spirituality (*β* = 0.36, *p* < 0.05) and academic values (*β* = 0.08, *p* < 0.05) at Wave 2 in a positive way. However, it did not significantly contribute to academic anxiety at Wave 2 (*β* = −0.03, *p* = 0.11). Prediction from spirituality at Wave 2 to academic values at Wave 2 (*β* = 0.61, *p* < 0.05) was positive, but negative for academic anxiety (*β* = −0.16, *p* < 0.05) (see Figure 2).

Table 3 indicates significant indirect effect of spirituality in the family functioning – academic values (*β* = 0.219, *p* < 0.05; 95% CI = 0.195 to 0.243) and family functioning – academic anxiety linkages (*β* = −0.057, *p* < 0.05; 95% CI = −0.071 to −0.043). Kindly note that the effect of family functioning to academic values after controlling for spirituality (*β* = 0.084, *p* < 0.05) was smaller than those without involving spirituality (*β* = 0.303, *p* < 0.05), and hence the mediating effect of spirituality was partial (see Baron and Kenny, 1986). Nevertheless, the effect of family functioning to academic anxiety was non-significant after controlling for spirituality and thus the mediation was full. Nearly three quarters of the total effect was explained by the indirect effect for academic values and academic anxiety of adolescents. Overall, the mediation model with spirituality as the mediator explained greater variances in academic values at Wave 2 (46.7%) than the direct effect model without including spirituality (11.0%). As such, Hypotheses 1, 3, 4, and 6 were evident. In addition, the mediation model with spirituality as the mediator explained higher variances in academic anxiety (3.9%) than the direct effect model without having spirituality (0.7%). The findings provide support for Hypotheses 2, 5 and 7.

**Table 3:** Results of mediation analyses in an overall SEM model (*N* = 4,922).

Paths	Standardized effects	95% CI (Lower bound)	95% CI (Upper bound)	% of indirect effect in the total effect	*R*^2^ (%)
*Academic values*					
Direct effects					
Family functioning ➔ spirituality	0.360	0.315	0.405	72.3	46.7
Spirituality ➔ academic values	0.609	0.585	0.633		
Family functioning ➔ academic values	0.084	0.051	0.117		
Indirect effect					
Family functioning ➔ spirituality ➔ academic values	0.219	0.195	0.243		
Total effect					
Family functioning ➔ academic values	0.303	0.266	0.340		
*Academic anxiety*					
Direct effects					
Family functioning ➔ spirituality	0.360	0.315	0.405	67.1	3.9
Spirituality ➔ academic anxiety	−0.159	−0.183	−0.135		
Family functioning ➔ academic anxiety	−0.028	−0.063	0.007		
Indirect effect					
Family functioning ➔ spirituality ➔ academic anxiety	−0.057	−0.071	−0.043		
*Total effect*					
Family functioning ➔ academic anxiety	−0.085	−0.118	−0.052		

The parameter estimate is significant when the confidence interval (CI) does not include 0 (*p* < 0.05).

## Discussion

The present investigation is a pioneer study to explore personal (spirituality) and contextual antecedents (family functioning) of academic values and academic anxiety of Chinese adolescents. Most importantly, it constitutes a theoretical advance to illustrate the mutual influence of family functioning and spirituality on both academic values and academic anxiety of Chinese adolescents. Besides, the present study has several methodological advances. First, this study is more holistic, unlike past research which only covered one aspect of family functions like parental involvement (e.g., Bi et al., 2020) and parent–child relationship (e.g., Ma et al., 2021). Second, longitudinal relationships among family functioning and spirituality, and academic values and academic anxiety of adolescents are rarely explored in China. As such, we utilized a large sample from China to examine such relationships.

In this study, we found that academic values of adolescents at Wave 2 were increased with their perceived family functioning at Wave 1, thus affirming the first hypothesis. The role of family as a significant social context for the development of academic values of adolescents is accentuated (e.g., Puente, 2022). Moreover, it echoes Eccles et al.’s (1983) expectancy-value model of achievement motivation which highlights the vital role of family processes in shaping adolescent academic values. As stated by Šimunović et al. (2018), parents are important socializers to their children. They would convey their academic-related values and expectations to their children and, subsequently affects their subjective intrinsic and utility values about learning. Rozek et al. (2015) advocates that transmission of intrinsic and utility values-beliefs from parents to their children rests heavily on parental behaviors such as encouragement, provision of educational resources and coactivity. However, since family functioning has been conceptualized in terms of only one dimension, such as family support (e.g., Puente, 2022) or parental involvement (e.g., Bi et al., 2020) in previous studies, it is assessed based on multiple domains of family interaction and parenting in this study, hence constituting a methodological innovation. This approach aligns with multi-dimensional conceptualization of family functioning including parenting style and interaction patterns (Yeung, 2016).

Besides, the total effect of family functioning on academic anxiety of Chinese adolescents was significantly negative in this study, thus supporting Hypothesis 2. This finding is consistent with previous research with Chinese adolescents (e.g., Ma et al., 2021; Chen et al., 2023). Also, it supports the thesis that family offers an important basis for healthy development of adolescents (e.g., Li et al., 2022). In addition, it is consistent with Peleg-Popko and Klingman’s (2002) family environment model of test anxiety that positive family interaction and authoritative parenting style would help adolescents reduce their academic anxiety. As stated by Ma et al. (2021), warm and supportive parents would provide sufficient freedom and guidance for children to handle their learning difficulties, and hence reduce their academic anxiety.

For the third research question, our findings revealed that spirituality was positively correlated with family functioning of Chinese adolescents over time, this supporting Hypothesis 3. This finding aligns with the notion by King and Boyatzis (2015) that parental support and care, and effective communication would facilitate spirituality development of adolescents via parent–child discussion on spiritual issues and role-modeling of spiritual attitudes and behaviors to their children. Moreover, it supports relational developmental systems theory of spiritual development which highlights the significance of multiple social contexts such as the family in promoting adolescents’ spirituality (King and Boyatzis, 2015). In fact, spirituality is one of the 15 important youth attributes in positive youth development attributes construct (Shek and Ma, 2010), which has been found to be associating with many favorable developmental outcomes of Chinese youth such as life satisfaction (Zhou et al., 2020) and academic well-being (Shek and Chai, 2020).

Besides, our finding on positive contribution of spirituality to academic values of Chinese adolescents supported Hypothesis 4. The finding echoes with Demerouti et al.’s (2001) Job Demands-Resources model which advocates that spirituality is a valuable personal resource to adolescents and it would motivate adolescents to strive for life meaning and academic excellence (Ariani, 2021). This motivational influence may be attributed to the fact that spirituality would cause individuals to feel comfortable and inspire them to try their best to do well in their study (Imron et al., 2023). It also echoes the findings by Ariani’s (2021) study which support the fact that the influence of spirituality on academic performance of students is mediated by intrinsic values of academic tasks.

With reference to the fifth research question, our findings support Hypothesis 5 that spirituality inversely predicted academic anxiety of Chinese adolescents over time. This finding supports the thesis that spirituality is important for the development of adolescent well-being (e.g., Nimmi et al., 2021). It is consistent with Lazarus and Folkman’s (1984) theory of stress and coping that spirituality serves as a coping resource to assist adolescents in overcoming their academic anxiety. Specifically, as stated by Ekwonye et al. (2020), adolescents would engage in some spiritual practices such as self-reflection and mediation to eliminate negative emotions surrounding academic-stress circumstances and analyze the stressful situation in great detail. Since past research has mostly investigated the impact of spirituality on anxiety of adolescents in the clinical setting (e.g., Vazifeh doust et al., 2020; Blum et al., 2021), the present finding enriches the literature on the relationship between spirituality and anxiety of adolescents in the “normal” academic setting.

Lastly, the mediating effect of spirituality on the links from family functioning to academic values and academic anxiety of adolescents was found (Hypothesis 6 and Hypothesis 7). First, spirituality partially mediated the influence of family functioning on academic values of adolescents over time, partially supporting Hypothesis 6, supporting Bronfenbrenner’s (1974) ecological theory that child developmental outcomes including academic values and academic anxiety are determined by mutual influence between personal (spirituality) and contextual (family functioning) factors. In addition, the present findings revealed that the impact of family functions on academic anxiety was fully mediated by spirituality of adolescents, supporting Hypothesis 7. This finding further highlights the significance of dynamic interplay between individual and contextual factors on adolescent development (Shahina and Parveen, 2020; Ariani, 2021). As a consequence, our findings are pioneering and worthy. Consistent with Shek et al.’s (2022b) findings, the present study advocates that the extent of the mediation exerted by positive youth development attributes like spirituality depends the type of the outcome variables.

### Implications

Theoretically, the significance of family functioning as a promoting factor in academic values and as a protective shield for academic anxiety of adolescents is highlighted. Although different theories (Cox and Paley, 1997; Peleg-Popko and Klingman, 2002) advocate the significance of family influence on academic-related outcomes of adolescents such as academic anxiety, research findings in this area are inconsistent (e.g., Song et al., 2015; Raymo et al., 2019). The present findings based on longitudinal data and a large sample empirically support an inverse relationship between family functioning and academic anxiety of adolescents which, hence supports family theories of academic anxiety (e.g., Conger et al., 1994; Peleg-Popko and Klingman, 2002). Moreover, our past papers using the same dataset has illustrated that the influence of family functions on delinquency of Chinese adolescents (Shek et al., 2022a). Since academic success helps reduce adolescent delinquency (e.g., Hoffmann, 2020), the present findings may assist us in developing more comprehensive understanding on the impact of positive family functioning on the prevention of delinquent behaviors in Chinese adolescents.

That is, favorable family functioning may help preventing adolescent delinquency through the promotion of academic values and success of adolescents. In short, this study broadens our horizon about the influence of family functioning and spirituality on academic-related outcomes of Chinese adolescents.

Regarding the practical implication, the present findings advocate the significance of family functions and adolescents’ spirituality in promoting academic values and reducing academic anxiety of adolescents via specialized intervention programs. As Chinese parents may actively involve in learning and eager to promote academic success of their children by offering different types of support like proper learning environment and resources (Huang and Gove, 2015; Li et al., 2021), our findings suggest that interventions targeted at promoting parent–child interactions and effective parenting styles, such as Juffer et al.’s (2023) positive parenting program, would enhance academic values of adolescents. Besides, as Chinese students may view the examination as threatening in their learning process and, hence develop academic anxiety (Lei et al., 2021). Again, the present findings advocate that interventions targeted at reducing maladaptive parental expectation and beliefs and promoting parent–child communication, such as Family Cognitive-Behavioral Therapy and Attachment-Based Family Therapy, would reduce academic anxiety of adolescents (see Peris et al., 2021).

Moreover, our findings suggest that interventions aimed to enhance spirituality of adolescents, such as Spiritual Group Training and Service Learning, would increase academic values and reduce academic anxiety of adolescents (Wirawan et al., 2018; Pong, 2023). Since spirituality is regarded as a positive youth development attribute, our findings echo those by Shek et al. (2022a,b) which illustrated the significance of positive youth development to young people. As stated by Zhu and Shek (2020), Tin Ka Ping P.A.T.H.S. Program was effective to enhance PYD attributes and lessen delinquency of adolescents in mainland China. As such, the Program could be adopted to enhance PYD attributes including spirituality of adolescents in China.

### Limitations

Nevertheless, there are several limitations of this study. Our findings were based on two waves of data only. To fully understand the causal impact of the mediator, more waves of data in a longer time span are recommended to collect in future research. Besides, generalization of present findings to other Chinese communities is difficult as we only collected and analyzed the responses from adolescents in Sichuan, China solely. To enhance the validity of the present findings, replication of this study in other Chinese communities is recommended. Last, a systematic bias might be introduced to the study because only self-reported data were gathered and analyzed. Collection of data through multiple sources such as parent and teacher ratings on adolescents’ anxiety is recommended.

## Data availability statement

The raw data supporting the conclusions of this article will be made available by the authors, without undue reservation.

## Ethics statement

The studies involving humans were approved by Ethics Research Committee of Sichuan University. The studies were conducted in accordance with the local legislation and institutional requirements. Written informed consent for participation in this study was provided by the participants’ legal guardians/next of kin.

## Author contributions

DS: Conceptualization, Funding acquisition, Investigation, Methodology, Project administration, Supervision, Writing – review & editing. KL: Formal analysis, Validation, Writing – original draft, Writing – review & editing. XL: Data curation, Project administration, Writing – review & editing. DD: Conceptualization, Methodology, Writing – review & editing. XZ: Conceptualization, Methodology, Writing – review & editing.

## References

[ref1] Adams S. K. Mushkat Z. Minkel J. (2022). Examining the moderator role of sleep quality in the relationship among test anxiety, academic success and mood. Psychol. Rep. 125, 2400–2415. doi: 10.1177/00332941211025268, PMID: 34134557

[ref2] Allen K.-A. Cordoba B. G. Ryan T. Arslan G. Slaten C. D. Ferguson J. K.. (2023). Examining predictors of school belonging using a socio-ecological perspective. J. Child Fam. Stud. 32, 2804–2819. doi: 10.1007/s10826-022-02305-1

[ref3] Ariani D. W. (2021). The role of religiosity and spirituality in motivating and improving students’ performance in Indonesia. J. Educ. Soc. Behav. Sci. 34, 52–63. doi: 10.9734/JESBS/2021/v34i830351

[ref4] Asendorpf J. B. van de Schoot R. Denissen J. J. A. Hutteman R. (2014). Reducing bias due to systematic attrition in longitudinal studies: the benefits of multiple imputation. Int. J. Behav. Dev. 38, 453–460. doi: 10.1177/0165025414542713

[ref5] Baron R. M. Kenny D. A. (1986). The moderator-mediator variable distinction in social psychological research: conceptual, strategic, and statistical considerations. J. Pers. Soc. Psychol. 51, 1173–1182. doi: 10.1037/0022-3514.51.6.1173, PMID: 3806354

[ref6] Bi X. Zhang L. Yang Y. Zhang W. (2020). Parenting practices, family obligation, and adolescents’ academic adjustment: cohort differences with social change in China. J. Res. Adolesc. 30, 721–734. doi: 10.1111/jora.12555 32109342

[ref7] Blum H. Rutt C. Nash C. Joyce V. Buonopane R. (2021). Mindfulness meditation and anxiety in adolescents on an inpatient psychiatric unit. J. Health Care Chaplain. 27, 65–83. doi: 10.1080/08854726.2019.1603918, PMID: 31021310

[ref9] Bronfenbrenner U. (1974). Developmental research, public policy, and the ecology of childhood. Child Dev. 45, 1–5. doi: 10.2307/1127743

[ref10] Brown T. A. (2006). Confirmatory factor analysis for applied research. New York: Guilford Press.

[ref11] Cassady J. C. (2022). “Anxiety in the schools: causes, consequences, and solutions for academic anxieties” in Handbook of stress and academic anxiety. eds. Gonzaga L. da Silva B. A. (Cham: Springer).

[ref12] Chen C. Liu P. Wu F. Wang H. Chen S. Zhang Y.. (2023). Factors associated with test anxiety among adolescents in Shenzhen. China. J. Affect. Disord. 323, 123–130. doi: 10.1016/j.jad.2022.11.048, PMID: 36427651

[ref13] Cheung G. W. Cooper-Thomas H. D. Lau R. S. Wang L. C. (2023). Reporting reliability, convergent and discriminant validity with structural equation modeling: a review and best-practice recommendations. Asia Pac. J. Manag. doi: 10.1007/s10490-023-09871-y

[ref14] Coleman J. S. (1988). Social capital in the creation of human capital. Am. J. Sociol. 94, S95–S120. doi: 10.1086/228943

[ref15] Conger R. D. Ge X. Elder G. H. Lorenzo F. O. Simons R. L. (1994). Economic stress, coercive family process, and developmental problems of adolescent. Child Dev. 65, 541–561. doi: 10.2307/1131401 8013239

[ref16] Cooper L. J. Valenti E. Laster-Loftus A. (2020). The role of spirituality in academic achievement. J. Instruct. Res. 9, 5–13.

[ref17] Cox M. J. Paley B. (1997). Families as systems. Annu. Rev. Psychol. 48, 243–267. doi: 10.1146/annurev.psych.48.1.243 9046561

[ref18] David R. Singh S. Ribeiro N. Gomes D. R. (2022). Does spirituality influence happiness and academic performance? Religions 13:617. doi: 10.3390/rel13070617

[ref19] Demerouti E. Bakker A. B. Nachreiner F. Schaufeli W. B. (2001). The job demands-resources model of burnout. J. Appl. Psychol. 86, 499–512. doi: 10.1037/0021-9010.86.3.499 11419809

[ref20] Dou D. Shek D. T. L. (2021). Predictive effect of internet addiction and academic values on satisfaction with academic performance among high school students in mainland China. Front. Psychol. 12:797906. doi: 10.3389/fpsyg.2021.797906, PMID: 35069391 PMC8771361

[ref21] Dou D. Shek D. T. L. Zhu X. Zhao L. (2021). Dimensionality of the Chinese CES-D: is it stable across gender, time, and samples? Int. J. Environ. Res. Public Health 18:11818. doi: 10.3390/ijerph182211818, PMID: 34831573 PMC8625664

[ref22] Eccles J. Adler T. F. Futterman R. Goff S. B. Kaczala C. M. Meece J. L.. (1983). “Expectancies, values, and academic behaviors” in Achievement and achievement motivation. ed. Spence J. T. (San Francisco, CA: W. H. Freeman), 75–146.

[ref24] Ekwonye A. U. Sheikhomar N. Phung V. (2020). Spirituality: a psychological resource for managing academic-related stressors. Ment. Health Relig. Cult. 23, 826–839. doi: 10.1080/13674676.2020.1823951

[ref25] Finney S. J. DiStefano C. (2006). “Non-normal and categorical data in structural equation modeling” in Structural equation modeling: A second course. eds. Hancock G. R. Mueller R. O. (Greenwich: Information Age Publishing).

[ref26] Folk J. B. Brown L. K. Marshall B. D. L. Ramos L. M. C. Gopalakrishnan L. Koinis-Mitchell D.. (2020). The prospective impact of family functioning and parenting practices on court-involved youth’s substance use and delinquent behavior. J. Youth Adolesc. 49, 238–251. doi: 10.1007/s10964-019-01099-8, PMID: 31399895 PMC7321799

[ref27] Govindarajoo M. V. Selvarajoo N. D. Ali M. S. (2022). Factors contributing to poor academic achievement among low performing pupils: a case study. Asian J. Univ. Educ. 18, 981–997. doi: 10.24191/ajue.v18i4.20008

[ref28] Guo J. Marsh H. W. Parker P. D. Morin A. J. S. Dicke T. (2017). Extending expectancy-value theory predictions of achievement and aspirations in science: dimensional comparison processes and expectancy-by-value interactions. Learn. Instr. 49, 81–91. doi: 10.1016/j.learninstruc.2016.12.007

[ref29] Hayek J. Schneider F. Lahoud N. Tueni M. de Vries H. (2022). Authoritative parenting stimulates academic achievement, also partly via self-efficacy and intention towards getting good grades. PLoS One 17:e0265595. doi: 10.1371/journal.pone.0265595, PMID: 35353817 PMC8967044

[ref30] Hill P. C. Pargament K. I. (2003). Advances in the conceptualization and measurement of religion and spirituality: implications for physical and medical health research. Am. Psychol. 58, 64–74. doi: 10.1037/0003-066X.58.1.64, PMID: 12674819

[ref31] Hoffmann J. P. (2020). Academic underachievement and delinquent behavior. Youth Soc. 52, 728–755. doi: 10.1177/0044118X18767035

[ref32] Holmes S. E. (2023). A qualitative inquiry into Christian faith transmission in the family context. Pract. Theol. 16, 604–617. doi: 10.1080/1756073X.2023.2184152

[ref33] Huang G. H.-C. Gove M. (2015). “Confucianism, Chinese families, and academic achievement: exploring how Confucianism and Asian descendant parenting practices influence children’s academic achievement” in Science education in East Asia: Pedagogical innovations and research-informed practices. ed. Khine M. S. (Netherlands: Springer).

[ref34] Imron I. Mawardi I. Şen A. (2023). The influence of spirituality on academic engagement through achievement motivation and resilience. Int. J. Islam. Educ. Psychol. 4:326. doi: 10.18196/ijiep.v4i2.19428

[ref35] Jia J. Wang L.-l. Xu J.-b. Lin X.-h. Zhang B. Jiang Q. (2021). Self-handicapping in Chinese medical students during the COVID-19 pandemic: the role of academic anxiety, procrastination and hardiness. Front. Psychol. 12:741821. doi: 10.3389/fpsyg.2021.741821, PMID: 34603160 PMC8484870

[ref36] Jiang S. Liu R. Ding Y. Fu X. Sun Y. Jiang R.. (2020). Implicit theories and engagement in math among Chinese adolescent students: a moderated mediation model of intrinsic value and academic self-efficacy. Front. Psychol. 11:1325. doi: 10.3389/fpsyg.2020.01325, PMID: 32676046 PMC7333438

[ref37] Jin X. (2023). Predicting academic success: machine learning analysis of student, parental, and school efforts. Asia Pacific Educ. Rev., 1–22. doi: 10.1007/s12564-023-09915-4

[ref38] Juffer F. Bakermans-Kranenburg M. J. Van Ijzendoorn M. H. (2023). Promoting positive parenting: An attachment-based intervention. New York: Routledge.

[ref39] Katrin A. A. Isabelle S. Franzis P. (2019). Longitudinal relations among self-concept, intrinsic value, and attainment value across secondary school years in three academic domains. J. Educ. Psychol. 111, 663–684. doi: 10.1037/edu0000313

[ref40] King P. E. Boyatzis C. J. (2015). “Religious and spiritual development” in Handbook of child psychology and developmental science: Socioemotional processes. eds. Lamb M. E. Lerner R. M. (New Jersey: John Wiley & Sons, Inc.).

[ref41] Lau R. S. Cheung G. W. (2012). Estimating and comparing specific mediation effects in complex latent variable models. Organ. Res. Methods 15, 3–16. doi: 10.1177/1094428110391673

[ref42] Lazarus R. S. Folkman S. (1984). Stress, appraisal, and coping. New York: Springer.

[ref43] Lee S. Jirásek I. Veselský P. Jirásková M. (2019). Gender and age differences in spiritual development among early adolescents. Eur. J. Dev. Psychol. 16, 680–696. doi: 10.1080/17405629.2018.1493990

[ref44] Lei W. Zhang H. Deng W. Wang H. Shao F. Hu W. (2021). Academic self-efficacy and test anxiety in high school students: a conditional process model of academic buoyancy and peer support. Sch. Psychol. Int. 42, 616–637. doi: 10.1177/01430343211039265

[ref47] Li J. Wang J. Li J.-Y. Qian S. Ling R.-Z. Jia R.-X.. (2022). Family socioeconomic status and mental health in Chinese adolescents: the multiple mediating role of social relationships. J. Public Health 44, 823–833. doi: 10.1093/pubmed/fdab280, PMID: 36455610

[ref48] Lin Y. Chen Y. Zhang Y. (2021). A study of the relationship between achievement goal orientation on online academic procrastination among junior high school students: multiple mediation analysis of task value and motivational regulation. Available at: https://doi-org.ezproxy.lb.polyu.edu.hk/10.1145/3474995.3475032 (Accessed September 23, 2023)

[ref49] Little T. D. (2013). Longitudinal structural equation modeling. New York, NY: The Guilford Press.

[ref50] Liu Y. Hau K.-T. Liu H. Wu J. Wang X. Zheng X. (2020). Multiplicative effect of intrinsic and extrinsic motivation on academic performance: a longitudinal study of Chinese students. J. Pers. 88, 584–595. doi: 10.1111/jopy.12512 31498427

[ref46] Li X. Huebner E. S. Tian L. (2021). Relations between achievement task values and academic achievement and depressive symptoms in Chinese elementary school students: variable-centered and person-centered perspectives. Sch. Psychol. 36, 167–180. doi: 10.1037/spq0000384, PMID: 34014699

[ref51] Lubis R. Hasanuddin H. (2023). The role of positive youth development as a mediator of the relationship between family function and lecturer-student relationship. Psympathic: Jurnal Ilmiah Psikologi 10, 29–38. doi: 10.15575/psy.v10i1.22733

[ref52] Ma M. Li D. Zhang L. (2021). Longitudinal prediction of children’s math anxiety from parent-child relationships. Learn. Individ. Differ. 88:102016. doi: 10.1016/j.lindif.2021.102016

[ref53] Meng X. Hu Z. (2023). The relationship between student motivation and academic performance: the mediating role of online learning behavior. Qual. Assur. Educ. 31, 167–180. doi: 10.1108/QAE-02-2022-0046

[ref54] Nimmi P. M. Binoy A. K. Joseph G. Suma R. (2021). Significance of developing spirituality among management students: discerning the impact on psychological resources and wellbeing. J. Appl. Res. High. Educ. 14, 317–331. doi: 10.1108/JARHE-10-2020-0372

[ref55] Özdil S. Ö. Kutlu Ö. (2019). Investigation of the mediator variable effect using BK, sobel and bootstrap methods (mathematical literacy case). Int. J. Progress. Educ. 15, 30–43. doi: 10.29329/ijpe.2019.189.3

[ref56] Peleg-Popko O. Klingman A. (2002). Family environment, discrepancies between perceived actual and desirable environment, and children’s test and trait anxiety. Br. J. Guid. Counc. 30, 451–466. doi: 10.1080/0306988021000025646

[ref57] Peris T. S. Thamrin H. Rozenman M. S. (2021). Family intervention for child and adolescent anxiety: a meta-analytic review of therapy targets, techniques, and outcomes. J. Affect. Disord. 286, 282–295. doi: 10.1016/j.jad.2021.02.053 33756306

[ref9001] Pong H. K. (2023). Contributions of service learning to the development of university students’ spiritual well-being and psychological health: a quasi-experimental study. J. Beliefs Values 44, 379–396. doi: 10.1080/13617672.2022.2133429

[ref58] Puente K. (2022). Examining the development of Latinx adolescents’ science intrinsic and utility values: a family systems approach. [dissertation’s thesis]. Irvine ProQuest Dissertations Publishing: University of California.

[ref59] Qin J. Ding Y. Gao J. Wu Y. Lv H. Wu J. (2021). Effects of COVID-19 on mental health and anxiety of adolescents aged 13–16 years: a comparative analysis of longitudinal data from China. Front. Psych. 12:695556. doi: 10.3389/fpsyt.2021.695556, PMID: 34354615 PMC8330831

[ref60] Raymo L. A. Somers C. L. Partridge R. T. (2019). Adolescent test anxiety: an examination of intraindividual and contextual predictors. School Ment. Health 11, 562–577. doi: 10.1007/s12310-018-09302-0

[ref61] Rozek C. S. Hyde J. S. Svoboda R. C. Hulleman C. S. Harackiewicz J. M. (2015). Gender differences in the effects of a utility-value intervention to help parents motivate adolescents in mathematics and science. J. Educ. Psychol. 107, 195–206. doi: 10.1037/a0036981

[ref62] Sales J. M. Irwin C. E. (2013). “A biopsychosocial perspective of adolescent health and disease” in Handbook of adolescent health psychology. eds. O’Donohue W. T. Benuto L. T. Tolle L. W. (New York: Springer Science+Business Media).

[ref63] Schweder S. Raufelder D. (2024). Does changing learning environments affect student motivation? Learn. Instr. 89:101829. doi: 10.1016/j.learninstruc.2023.101829

[ref64] Shahina G. Parveen A. (2020). Role of spirituality in building up resilience and mental health among adolescents. Ind. J. Posit. Psychol. Rev. 11, 392–397. doi: 10.15614/IJPP/2020/V11I4/207695

[ref67] Shek D. T. L. Chai C. W. Y. Dou D. (2021). Parenting factors and meaning of life among Chinese adolescents: a six-wave longitudinal study. J. Adolesc. 87, 117–132. doi: 10.1016/j.adolescence.2021.01.004, PMID: 33581398

[ref66] Shek D. T. L. Chai W. (2020). The impact of positive youth development attributes and life satisfaction on academic well-being: a longitudinal mediation study. Front. Psychol. 11:2126. doi: 10.3389/fpsyg.2020.02126, PMID: 32982869 PMC7490328

[ref69] Shek D. T. L. Leung K. H. Dou D. Zhu X. (2022a). Family functioning and adolescent delinquency in mainland China: positive youth development attributes as a mediator. Front. Psych. 13:883439. doi: 10.3389/fpsyt.2022.883439, PMID: 35573365 PMC9096017

[ref70] Shek D. T. L. Leung K. H. Dou D. Zhu X. (2022b). Impact of family functioning on adolescent materialism and egocentrism in mainland China: positive youth development (PYD) attributes as a mediator. Int. J. Environ. Res. Public Health 19:11038. doi: 10.3390/ijerph191711038, PMID: 36078755 PMC9517865

[ref71] Shek D. T. L. Leung K. H. Li X. Dou D. (2023). Psychometric properties of the Chinese family assessment instrument: evidence from mainland China. Front. Psychol. 14:1290224. doi: 10.3389/fpsyg.2023.1290224, PMID: 38152558 PMC10752606

[ref72] Shek D. T. L. Ma C. M. S. (2010). Dimensionality of the Chinese positive youth development scale: confirmatory factor analyses. Soc. Indic. Res. 98, 41–59. doi: 10.1007/s11205-009-9515-9

[ref65] Shek D. T. L. (2002). “Assessment of family functioning in Chinese adolescents: the Chinese family assessment instrument” in International perspectives on child and adolescent mental health. eds. Singh N. N. Ollendick T. Singh A. N. (London: Elsevier).

[ref73] Shroff D. M. Breaux R. von Suchodoletz A. (2023). Understanding the association between spirituality and mental health outcomes in adolescents in two non-Western countries: exploring self-control as a potential mediator. Dev. Psychopathol. 35, 1434–1443. doi: 10.1017/S0954579421001334, PMID: 34937591

[ref74] Šimunović M. Ercegovac I. R. Burušić J. (2018). How important is it to my parents? Transmission of STEM academic values: the role of parents’ values and practices and children’s perceptions of parental influences. Int. J. Sci. Educ. 40, 977–995. doi: 10.1080/09500693.2018.1460696

[ref75] Song J. Bong M. Lee K. Kim S.-i. (2015). Longitudinal investigation into the role of perceived social support in adolescents’ academic motivation and achievement. J. Educ. Psychol. 107, 821–841. doi: 10.1037/edu0000016

[ref77] Stewart S. L. Semovski V. Lapshina N. (2022). Adolescent inpatient mental health admissions: an exploration of interpersonal polyvictimization, family dysfunction, self-harm and suicidal behaviours. Child Psychiatry Hum. Dev. doi: 10.1007/s10578-022-01450-4, PMID: 36315373 PMC11245427

[ref78] Stokes C. E. (2008). The role of parental religiosity in high school completion. Sociol. Spectr. 28, 531–555. doi: 10.1080/02732170802206153, PMID: 20396644 PMC2854401

[ref79] Tamayo-Aguledo W. Acosta-Ortiz A. Hamid A. Gómez-García C. García-Durán M. C. Daccach-González V.. (2022). Family functioning but not social capital is associated with better mental health in adolescents affected by violence and displacement by armed conflict in Colombia. Int. J. Soc. Psychiatry 68, 1598–1606. doi: 10.1177/00207640211045417, PMID: 34496653 PMC9597145

[ref80] Trikoilis D. (2023). Investigating the factors affecting adolescents’ test anxiety in Greece during the COVID-19 pandemic. Pastor. Care Educ. 42, 125–145. doi: 10.1080/02643944.2023.2214920

[ref81] Tripon C. Gona I. Bulgac A. (2023). Nurturing minds and sustainability: an exploration of educational interactions and their impact on student well-being and assessment in a sustainable university. Sustain. For. 15:9349. doi: 10.3390/su15129349

[ref82] Urhahne D. Wijnia L. (2023). Theories of motivation in education: an integrative framework. Educ. Psychol. Rev. 35:45. doi: 10.1007/s10648-023-09767-9

[ref83] van Eickels R. L. Tsarpalis-Fragkoulidis A. Zemp M. (2022). Family cohesion, shame-proneness, expressive suppression, and adolescent mental health - a path model approach. Front. Psychol. 13:921250. doi: 10.3389/fpsyg.2022.921250, PMID: 35992453 PMC9382198

[ref84] Vazifeh doust M. Hojjati H. Farhangi H. (2020). Effect of spiritual care based on Ghalbe Salim on anxiety in adolescent with cancer. J. Relig. Health 59, 2857–2865. doi: 10.1007/s10943-019-00869-9, PMID: 31240515

[ref85] Wahyuni E. N. Aziz R. Mangestuti R. (2020). Family, spirituality, and mental health in higher education. In Proceedings of the 3rd International Conference on Psychology in Health, Educational, Social, and Organizational Settings - ICP-HESOS SciTePress.

[ref86] Wang Y. Rocabado G. A. Lewis J. E. Lewis S. E. (2021). Prompts to promote success: evaluating utility value and growth mindset interventions on general chemistry students’ attitude and academic performance. J. Chem. Educ. 98, 1476–1488. doi: 10.1021/acs.jchemed.0c01497

[ref87] Weston R. Gore P. A. (2006). A brief guide to structural equation modeling. Couns. Psychol. 34, 719–751. doi: 10.1177/0011000006286345

[ref89] Wirawan H. Jufri M. Anto Patak A. (2018). Spiritual group training for adolescences: investigating the effect of group training on spiritual well-being. Int. J. Less. Learn. Stud. 7, 62–74. doi: 10.1108/IJLLS-10-2016-0040

[ref90] Wu Y. Xie F. Jiang R. (2022). Academic anxiety, self-regulated learning ability, and self-esteem in Chinese candidates for college entrance examination during the COVID-19 outbreak: a survey study. Psychol. Res. Behav. Manag. 15, 2383–2390. doi: 10.2147/PRBM.S360127, PMID: 36062032 PMC9438794

[ref91] Xie F. Xin Z. Chen X. Zhang L. (2019). Gender difference of Chinese high school students’ math anxiety: the effects of self-esteem, test anxiety and general anxiety. Sex Roles 81, 235–244. doi: 10.1007/s11199-018-0982-9

[ref92] Xu L. Wang Z. Tao Z. Yu C. (2022). English-learning stress and performance in Chinese college students: a serial mediation model of academic anxiety and academic burnout and the protective effect of grit. Front. Psychol. 13:1032675. doi: 10.3389/fpsyg.2022.1032675 36533059 PMC9749891

[ref93] Xu X. Xia M. Pang W. (2021). Family socioeconomic status and Chinese high school students’ test anxiety: serial mediating role of parental psychological control, learning resources, and student academic self-efficacy. Scand. J. Psychol. 62, 689–698. doi: 10.1111/sjop.12750, PMID: 34155654

[ref94] Yeung J. W. K. (2016). Parent-child discrepant effects on positive youth outcomes at the aggregate family functioning context in Hong Kong. Appl. Res. Qual. Life 11, 871–890. doi: 10.1007/s11482-015-9404-0

[ref95] Yeung S. S. S. King R. B. Nalipay M. J. N. Cai Y. (2022). Exploring the interplay between socioeconomic status and reading achievement: an expectancy-value perspective. Br. J. Educ. Psychol. 92, 1196–1214. doi: 10.1111/bjep.12495, PMID: 35243608

[ref97] Yuan R. (2023). Chinese university EFL learners’ foreign language classroom anxiety and enjoyment in an online learning environment during the COVID-19 pandemic. Asia Pac. J. Educ., 1–17. doi: 10.1080/02188791.2023.2165036

[ref96] Yu L. Shek D. T. L. (2021). Positive youth development attributes and parenting as protective factors against adolescent social networking addiction in Hong Kong. Front. Pediatr. 9:649232. doi: 10.3389/fped.2021.649232, PMID: 33816410 PMC8012543

[ref98] Yusdiana F. H. Hitipeuw I. Chusniyah T. (2019). Fear of failure: the paranoia of academically gifted students. Int. J. Sci. Technol. Res. 8, 3300–3307.

[ref99] Zanabazar A. Deleg A. Ravdan M. (2023). A study of factors causing math anxiety among undergraduate students. Int. J. Innov. Res. Sci. Stud. 6, 578–585. doi: 10.53894/ijirss.v6i3.1609

[ref100] Zeng Y. Zhang J. Wei J. Li S. (2022). The impact of undergraduates’ social isolation on smartphone addiction: the roles of academic anxiety and social media use. Int. J. Environ. Res. Public Health 19:15903. doi: 10.3390/ijerph192315903, PMID: 36497974 PMC9738847

[ref101] Zhou Z. Shek D. T. L. Zhu X. (2020). The importance of positive youth development attributes to life satisfaction and hopelessness in mainland Chinese adolescents. Front. Psychol. 11:553313. doi: 10.3389/fpsyg.2020.553313 33101126 PMC7554621

[ref102] Zhu X. Shek D. T. L. (2020). Impact of a positive youth development program on junior high school students in mainland China: a pioneer study. Child Youth Serv. Rev. 114:105022. doi: 10.1016/j.childyouth.2020.105022

[ref103] Zhu Y. Wang S. Shi X. (2022). Poor sleep quality mediates the relationship between intra-family conflict and mental health problems in Chinese adolescents: a three-wave longitudinal study. Curr. Psychol. 42, 25696–25705. doi: 10.1007/s12144-022-03700-z

